# Epidemiology and risk factors in CKD patients with pulmonary hypertension: a retrospective study

**DOI:** 10.1186/s12882-018-0866-9

**Published:** 2018-03-20

**Authors:** Qian Zhang, Le Wang, Hongbing Zeng, Yongman Lv, Yi Huang

**Affiliations:** Division of Nephrology, Department of Internal Medicine, Tongji Hospital, Tongji Medical College, Huazhong University of Science and Technology, Wuhan, Hubei People’s Republic of China

**Keywords:** Pulmonary hypertension, Chronic kidney disease, Prevalence, Risk factors

## Abstract

**Background:**

Pulmonary hypertension (PH) is a rare disease often associated with high mortality and is recently recognized as a common complication secondary to chronic kidney disease (CKD). Epidemiological data for this disorder across the spectrum of CKD is poorly understood.

**Methods:**

We retrospectively analyzed 705 CKD patients with complete clinical records from July 2013 to September 2015. All the patients were estimated by echocardiography and PH was defined as pulmonary artery systolic pressure (PASP) > 35 mmHg. The prevalence of PH in CKD patients was investigated, and their association was evaluated with a logistic regression model.

**Results:**

The overall prevalence of PH was 47.38%, in which mild, moderate and severe PH accounted for 22.13, 15.04 and 10.21%, respectively. The prevalence of PH in CKD stage 1–5 was 14.29, 33.33, 38.89, 40.91 and 64.47%. The prevalence of total PH was 57.63% in PD patients and 58.82% in HD patients. Compared with the non-dialysis patients, the prevalence of PH was much higher in patients receiving dialysis. Body mass index (BMI), hemoglobin, triglyceride (TG), proteinuria, parathyroid hormone (PTH) and estimated glomerular filtration rate (eGFR) were independent risk factors of PH in CKD patients.

**Conclusions:**

The prevalence of PH is increased with the deterioration of renal function, however, which has no direct relation to the severity of PH. PH occurs more frequently in dialysis patients. Higher BMI and TG, more sever anemia, proteinuria and secondary hyperparathyroidism, poor renal dysfunction predict predict the more prevalence of PH in CKD patients.

## Background

Pulmonary hypertension (PH) is primarily a disease of the small arteries of the pulmonary vasculature, with progressive obliteration leading to the increases in pulmonary vascular resistance (PVR) and pulmonary arterial pressure (PAP) that characterize the disease [1]. The increased PVR often leads to right ventricular failure associated with high mortality [2]. The mechanisms responsible for PH still remain incompletely understood. Many studies have shown that PH occurred frequently in patients with chronic kidney disease (CKD) and some of them have explored the relation between CKD and PH [3, 4]. PH in patients with CKD may be induced and aggravated by left ventricular disorders and risk factors typical of CKD, including volume overload, an arteriovenous fistula, sleep-disordered breathing, exposure to dialysis membranes, endothelial dysfunction, vascular calcification and stiffening, and severe anemia [3]. A review reported that the prevalence of PH ranged from 9–39% in individuals with stage 5 CKD, 18.8–68.8% in hemodialysis patients, and 0–42% in patients on peritoneal dialysis therapy [3]. However, few epidemiologic data are available for earlier stages of CKD. Based on a retrospective analysis of 705 CKD patients, this study aims to investigate the epidemiology and prevalence of PH in different stages of CKD and to determinate the related risk factors of PH in CKD.

## Methods

### Participants

The retrospective study was performed in the renal division of Tongji Hospital in China. All patients with CKD who received echocardiography were included from July 2013 to September 2015. The etiologies for CKD included glomerulonephritis, diabetic nephropathy, hypertensive nephropathy, lupus nephritis, polycystic kidney disease and others. The inclusion criteria were as follows: (1) The age of the patients was more than 18 years old; (2) All patients were diagnosed as CKD according to K/DOQI guidelines, including peritoneal dialysis (PD) and hemodialysis (HD) patients; (3) All patients had complete clinical data. Patients were excluded as follows: (1) Connective tissue disease except for lupus, HIV infection, congenital heart disease, acute heart failure, portal hypertension and pulmonary veno-occlusive disease, drugs and toxins; (2) COPD, interstitial lung disease and sleep apnea; (3) Obstructive of pulmonary arterial vessels by thromboemboli, tumors, or foreign bodies.

### Clinical and laboratory data collection

Patient data on baseline characteristics (age, gender, BMI, medication used), etiology of CKD, laboratory tests and hemodynamics were recorded. Two-dimensional (2D)-guided M-mode echocardiography were performed by experienced technicians with an Acuson Sequoia, C256 (Mountain View, CA, USA) ultrasound machine. A tricuspid systolic jet was recorded from the parasternal or apical window with the continuous wave Doppler probe. Pulmonary artery systolic pressure (PASP) was estimated by a modified Bernoulli equation: PASP = 4 × (tricuspid systolic jet)^2^ + 10 mmHg (estimated right atrial pressure) [5, 6]. PH was defined as a PASP > 35 mmHg [7, 8]. The severity of PH was categorized according to the SPAP as follows: mild (35–45 mmHg), moderate (45–60 mmHg) and severe (> 60 mmHg) [9].

### Data analysis

All statistical analysis was performed using the SPSS 18.0 software. The categorical data were reported as numbers and the differences were tested with the χ^2^ test. Continuous variables were summarized as means ± standard deviation (SD). Differences among groups were compared with Student’s *t* test for the normally distributed variables and Mann-Whitney *U* test for the non- normally distributed variables. Descriptive statistics related to the prevalence of PH were calculated with logistic regression analysis. The *P*-values reported were two-sided and taken to be significant at < 0.05.

## Results

From July 2013 to September 2015, CKD patients from our renal division who received echocardiography were recruited. A total of 705 patients satisfied the inclusion criteria. There were 400 males and 305 females with a mean age of 48.97 ± 16.74 years. Common cause of CKD included glomerulonephritis (68.37%), diabetic nephropathy (14.75%), hypertensive nephropathy (8.09%), lupus nephritis (1.84%), polycystic kidney disease (2.84%) and others (4.11%). The baseline demographic, clinical, laboratory and hemodynamic characteristics of the patients in different stages of CKD were summarized in Table 1.

**Table 1:** Baseline demographic, clinical, laboratory, and hemodynamic characteristics of the study population by eGFR

	All	CKD1	CKD2	CKD3	CKD4	CKD5
No. of patients	705	84	63	108	132	318
Age (years)	48.12 ± 15.02	39.50 ± 15.59	45.49 ± 15.10	49.52 ± 15.15	51.54 ± 15.30	46.44 ± 14.63
Gender						
Male, n (%)	390 (55.32%)	44 (52.38%)	25 (39.68%)	75 (69.44%)	85 (64.39%)	161 (52.83%)
Female, n (%)	315 (44.68%)	40 (47.62%)	38 (60.32%)	33 (30.56%)	47 (35.61%)	157 (47.17%)
BMI (kg/m^2^)	22.32 ± 4.45	21.88 ± 4.08	22.43 ± 4.58	22.04 ± 4.56	22.19 ± 3.88	22.56 ± 4.70
Etiology of CKD, n (%)						
Glomerulonephritis	481 (68.23%)	65 (77.38%)	39 (61.90%)	79 (73.15%)	83 (62.88%)	215 (67.61%)
Diabetic nephropathy	104 (14.75%)	9 (10.71%)	13 (20.63%)	12 (11.11%)	25 (18.94%)	45 (14.15%)
Hypertensive nephropathy	57 (8.09%)	4 (4.76%)	6 (9.52%)	9 (8.33%)	13 (9.85%)	25 (7.86%)
Lupus nephritis	14 (1.99%)	0 (0%)	1 (1.59%)	1 (0.93%)	5 (3.79%)	7 (2.20%)
Polycystic kidney disease	20 (2.84%)	3 (3.57%)	1 (1.59%)	3 (2.78%)	1 (0.76%)	12 (3.77%)
Others	29 (4.11%)	3 (3.57%)	3 (4.76%)	4 (3.70%)	5 (3.79%)	14 (4.40%)
Medications, n (%)						
ACEI or ARB	294 (41.70%)	49 (58.33%)	16 (25.40%)	0 (0%)	51 (38.64%)	178 (55.97%)
Diuretics	88 (12.48%)	0 (0%)	0 (0%)	28 (25.93%)	24 (18.18%)	36 (11.32%)
CCB	407 (57.73%)	21 (25%)	21 (25%)	64 (59.26%)	92 (69.70%)	209 (65.72%)
β-blocker	285 (40.43%)	13 (15.48%)	16 (25.40%)	43 (39.81%)	64 (48.48%)	149 (46.86%)
α-blocker	18 (2.55%)	0 (0%)	0 (0%)	2 (1.85%)	5 (3.79%)	11 (3.46%)
Digoxin	14 (1.99%)	0 (0%)	0 (0%)	0 (0%)	0 (0%)	14 (4.40%)
Warfarin	26 (3.69%)	0 (0%)	0 (0%)	12 (11.11%)	0 (0%)	14 (4.40%)
Prostacyclin	247 (35.04%)	0 (0%)	12 (19.05%)	76 (70.37%)	82 (62.12%)	77 (24.21%)
Laboratory tests						
Hb (g/L)	94.19 ± 23.46	103.66 ± 20.54	100.68 ± 21.29	103.05 ± 26.33	94.64 ± 23.56	87.22 ± 21.26
ALB (g/L)	34.75 ± 5.68	35.60 ± 5.07	34.39 ± 5.26	34.80 ± 5.59	34.27 ± 5.25	34.78 ± 6.11
TC (mmol/L)	4.37 ± 1.16	4.41 ± 0.98	4.50 ± 1.28	4.44 ± 1.30	4.12 ± 0.93	4.41 ± 1.21
TG (mmol/L)	1.68 ± 1.10	1.52 ± 0.82	1.53 ± 0.80	1.66 ± 1.03	1.82 ± 1.26	1.70 ± 1.17
Proteinuria (mg/24 h)	1783.13 ± 1233.31	1387.64 ± 1016.40	1586.44 ± 1122.68	1543.20 ± 1207.24	1699.50 ± 1158.92	2042.75 ± 1294.12
Ca (mmol/L)	2.17 ± 0.19	2.20 ± 0.11	2.16 ± 0.11	2.27 ± 0.12	2.22 ± 0.14	2.22 ± 0.24
P (mmol/L)	1.41 ± 0.52	0.99 ± 0.12	1.04 ± 0.13	1.11 ± 0.13	1.25 ± 0.23	1.76 ± 0.57
PTH (pg/ml)	326.20 ± 180.71	271.98 ± 123.31	334.10 ± 182.36	290.55 ± 153.09	320.75 ± 146.67	353.33 ± 207.99
Ferritin (ng/ml)	350.27 ± 192.52	319.08 ± 95.40	331.24 ± 92.25	366.82 ± 258.77	354.95 ± 132.96	354.72 ± 218.88
SI (μmol/L)	12.38 ± 5.62	12.74 ± 4.88	12.36 ± 5.62	12.49 ± 5.36	11.60 ± 5.69	12.58 ± 5.86
C3 (g/L)	0.96 ± 0.29	1.01 ± 0.32	0.96 ± 0.29	0.98 ± 0.31	0.93 ± 0.31	0.94 ± 0.27
Hemodynamics						
SBP (mmHg)	141.24 ± 14.14	137.43 ± 12.18	138.30 ± 9.89	138.45 ± 16.09	142.33 ± 14.78	143.32 ± 13.97
DBP (mmHg)	85.44 ± 9.80	82.41 ± 9.21	84.48 ± 8.34	84.43 ± 10.35	84.28 ± 10.08	87.26 ± 9.62
PASP (mmHg)	39.39 ± 13.86	32.40 ± 9.35	38.38 ± 14.66	38.04 ± 13.69	38.86 ± 13.78	42.13 ± 14.09
LVEF (%)	62.05 ± 10.04	65.08 ± 10.02	64.81 ± 6.49	63.92 ± 8.46	61.45 ± 9.88	60.31 ± 10.79

*eGFR* estimated glomerular filtration rate, *CKD* chronic kidney disease, *BMI* body mass index, *ACEI* angiotensin converting enzyme inhibitor, *ARB* angiotensin receptor blocker, *CCB* calcium channel blocker, *Hb* hemoglobin, *ALB* albumin, *TC* total cholesterol, *TG* triglyceride, *PTH* parathyroid hormone, *SI* serum iron, *SBP* systolic blood pressure, *DBP* diastolic blood pressure, *PASP* pulmonary artery systolic pressure, *LVEF* left ventricular ejection fraction. Results are reported as means ± standard deviation

### Epidemiology of PH in different stages of CKD

**Fig. 1:**
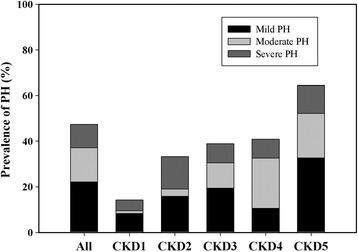
Prevalence of mild, moderate and severe PH in different stages of CKD patients

The overall prevalence of PH was 47.38% (334/705), in which mild, moderate and severe PH accounted for 22.13, 15.04 and 10.21%, respectively. The prevalence of PH in CKD stage 1–5 was 14.29% (12/84), 33.33% (21/63), 38.89% (42/108), 40.91% (54/132) and 64.47% (205/318) (Fig. 1). With the deterioration of renal function, the incidence of PH was increased gradually. However, the severity of PH seemed to have no regular distribution.

**Fig. 2:**
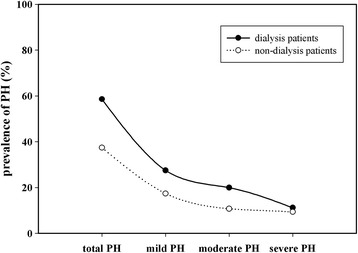
Prevalence of mild, moderate and severe PH in dialysis patients. Compared with the non-dialysis patients, the prevalence of PH was much higher in patients received dialysis

Among all the CKD patients, 331 ones have received dialysis. Compared with the non-dialysis patients, the prevalence of PH was much higher in patients received dialysis. In dialysis patients, mild-to-moderate PH appeared frequently. However, there was no difference in the incidence of severe PH between dialysis patients and non-dialysis patients (Fig. 2).

**Fig. 3:**
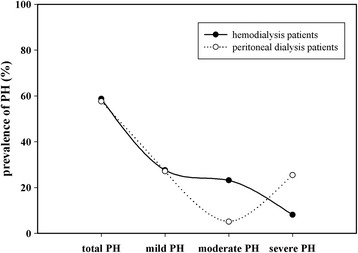
Prevalence of mild, moderate and severe PH in HD and PD patients

Among all the dialysis patients, there were 59 for PD and 272 for HD. The prevalence of PH was 57.63% in PD patients and 58.82% in HD patients. However, the incidence of PH in PD and HD patients was different according to the severity of PH. The prevalence of moderate and severe PH was 5.08 and 25.42% in PD patients, separately. However, the prevalence of moderate and severe PH was 23.16 and 8.09% in HD patients, separately. Interestingly, the incidence was no difference in mild PH between PD and HD patients (Fig. 3).

### Determinants of PH in CKD patients

The baseline demographic, clinical, laboratory, and hemodynamic characteristics of the study population by PH severity were shown in Table 2. Compared with the non-PH patients, the patients with PH had lower hemoglobin, albumin, complement 3, estimated glomerular filtration rate (eGFR), left ventricular ejection fraction (LVEF) levels and higher body mass index (BMI), triglyceride (TG), parathyroid hormone (PTH), proteinuria, blood pressure (systolic and diastolic) and time of dialysis. With PH as dependent variable and the above factors as covariates, only six variables, BMI, hemoglobin, TG, proteinuria, PTH and eGFR, were independent risk factors in the prevalence of PH in CKD patients. The multivariate determinants of prevalence of PH were shown in Table 3.

**Table 2:** Baseline demographic, clinical, laboratory, and hemodynamic characteristics of the study population by PASP

	All	PASP ≤35 mmHg	35 mmHg < PASP ≤45 mmHg	45 mmHg < PASP ≤60 mmHg	PASP > 60 mmHg
No. of patients	705	371	156	106	72
Age (years)	48.12 ± 15.02	48.43 ± 15.50	45.50 ± 14.08	49.83 ± 15.81	49.71 ± 12.63
Gender					
Male, n (%)	400	231 (57.75%)	73 (18.25%)	46 (11.50%)	40 (10.00%)
Female, n (%)	305	140 (45.90%)	83 (27.21%)^a^	60 (19.67%)^a^	32 (10.49%)
BMI (kg/m^2^)	22.32 ± 4.45	21.55 ± 3.96	22.98 ± 4.94^**^	22.47 ± 4.39	24.65 ± 4.85^***^
Etiology of CKD, n (%)					
Glomerulonephritis	481	268 (55.72%)	100 (20.79%)	69 (14.35%)	44 (9.15%)
Diabetic nephropathy	104	45 (43.27%)	31 (29.81%)^b^	17 (16.35%)	11 (10.58%)
Hypertensive nephropathy	57	30 (52.36%)	13 (22.81%)	9 (15.79%)	5 (8.77%)
Lupus nephritis	14	4 (28.57%)	0 (0%)	3 (21.43%)	7 (50.00%)^b^
Polycystic kidney disease	20	11 (55.00%)	6 (30.00%)	3 (15.00%)	0 (0%)
Others	29	13 (44.83%)	6 (20.69%)	5 (17.24%)	5 (17.24%)
No. of anti-hypertensives					
0 anti-hypertensive	72	41 (56.94%)	14 (19.44%)	9 (12.50%)	8 (11.11%)
1 anti-hypertensives	180	107 (59.44%)	28 (15.56%)	29 (16.11%)	16 (8.89%)
2 anti-hypertensives	230	132 (57.39%)	56 (24.35%)	22 (9.57%)	20 (8.70%)
3 anti-hypertensives	155	63 (40.65%)	39 (25.16%)	37 (23.87%)	16 (10.32%)
≥ 4 anti-hypertensives	68	28 (41.18%)	19 (27.94%)	9 (13.24%)	12 (17.65%)
Other medications, n (%)					
Digoxin	14	5 (35.71%)	0 (0%)	5 (35.71%)	4 (28.57%)
Warfarin	26	5 (19.23%)	12 (46.15%)	5 (19.23%)	4 (15.38%)
Prostacyclins	247	120 (48.58%)	55 (22.27%)	37 (14.98%)	35 (14.17%)
Laboratory tests					
Hb (g/L)	94.19 ± 23.46	107.45 ± 19.82	80.37 ± 19.50^***^	77.91 ± 19.40^***^	81.37 ± 12.77^***^
ALB (g/L)	34.75 ± 5.68	35.44 ± 5.11	34.50 ± 5.98	33.15 ± 6.92^*^	34.06 ± 5.31
TC (mmol/L)	4.37 ± 1.16	4.28 ± 0.99	4.43 ± 1.58	4.57 ± 1.10	4.38 ± 0.93
TG (mmol/L)	1.68 ± 1.10	1.52 ± 0.81	1.90 ± 1.50^*^	1.61 ± 0.95	2.13 ± 1.43^**^
Proteinuria (mg/24 h)	1783.13 ± 1233.31	1176.14 ± 971.05	2132.18 ± 1051.07^***^	2737.32 ± 1372.09^***^	2749.72 ± 687.34^***^
Ca (mmol/L)	2.17 ± 0.19	2.18 ± 0.18	2.17 ± 0.20	2.14 ± 0.20	2.21 ± 0.20
P (mmol/L)	1.41 ± 0.52	1.31 ± 0.46	1.51 ± 0.53^***^	1.57 ± 0.62^***^	1.43 ± 0.55
PTH (pg/ml)	326.20 ± 180.71	266.02 ± 148.51	358.54 ± 163.65^***^	353.40 ± 154.59^***^	526.15 ± 228.42^***^
Ferritin (ng/ml)	350.27 ± 192.52	354.34 ± 224.21	301.23 ± 142.90^**^	370.56 ± 146.29	405.68 ± 142.19
SI (μmol/L)	12.38 ± 5.62	12.46 ± 5.81	13.05 ± 6.51	11.50 ± 4.60	11.83 ± 3.35
C3 (g/L)	0.96 ± 0.29	1.01 ± 0.33	0.92 ± 0.26^**^	0.84 ± 0.22^***^	0.95 ± 0.21
Hemodynamics					
SBP (mmHg)	141.24 ± 14.14	138.46 ± 13.19	143.71 ± 16.22^**^	145.53 ± 14.98^***^	143.89 ± 9.00^***^
DBP (mmHg)	85.44 ± 9.80	83.59 ± 9.45	87.28 ± 10.89^**^	88.49 ± 10.08^***^	86.54 ± 6.08^**^
eGFR (ml/min/1.73m^2^)	33.55 ± 32.51	43.71 ± 36.13	22.84 ± 25.27^***^	18.48 ± 15.57^***^	26.57 ± 27.29^***^
LVEF (%)	62.05 ± 10.04	63.50 ± 9.33	66.13 ± 7.19^**^	56.23 ± 11.05^***^	54.26 ± 9.66^***^
No. of dialysis patients (%)	331	136 (36.66%)	81 (51.92%)	66 (62.26%)	48 (66.67%)
Time of dialysis (months)	16.89 ± 14.16	16.58 ± 14.01	15.54 ± 11.42^***^	16.48 ± 14.46^***^	20.29 ± 17.64^***^

*PASP* pulmonary artery systolic pressure, *CKD* chronic kidney disease, *BMI* body mass index, *ACEI* angiotensin converting enzyme inhibitor, *ARB* angiotensin receptor blocker, *CCB* calcium channel blocker, *Hb* hemoglobin, *ALB* albumin, *TC* total cholesterol, *TG* triglyceride, *PTH* parathyroid hormone, *SI* serum iron, *SBP* systolic blood pressure, *DBP* diastolic blood pressure, *eGFR* estimated glomerular filtration rate, *LVEF* left ventricular ejection fraction. Results are reported as means ± standard deviation. Compared with non-PH group, ^*^p < 0.05, ^**^p < 0.01, ^***^p < 0.001; Compared with male group, ^a^*p* < 0.05; Compared with glomerulonephritis group, ^b^*p* < 0.05

**Table 3:** Multivariate ORs for prevalence of PH in CKD patients

Determinants	OR (95%CI)	*P*-value
Gender	0.874 (0.541–1.412)	0.581
BMI (kg/m^2^)	1.070 (1.011–1.133)	0.020
Glomerulonephritis	reference	
Diabetic nephropathy	0.926 (0.299–2.869)	0.894
Hypertensive nephropathy	1.589 (0.451–5.597)	0.471
Lupus nephritis	0.542 (0.141–2.078)	0.372
Polycystic kidney disease	1.335 (0.137–13.056)	0.804
Others	0.753 (0.128–4.438)	0.754
Hb (g/L)	0.942 (0.929–0.954)	0.000
ALB (g/L)	1.017 (0.977–1.059)	0.411
TG (mmol/L)	1.445 (1.147–1.822)	0.002
Proteinuria (mg/24 h)	1.001 (1.001–1.001)	0.000
P (mmol/L)	0.826 (0.488–1.397)	0.475
PTH (pg/ml)	1.004 (1.002–1.005)	0.000
C3 (g/L)	0.496 (0.172–1.012)	0.064
SBP (mmHg)	1.003 (0.982–1.024)	0.785
DBP (mmHg)	1.021 (0.990–1.053)	0.180
eGFR (ml/min/1.73m^2^)	0.981 (0.971–0.990)	0.000
LVEF (%)	0.987 (0.962–1.012)	0.302
Time of dialysis (months)	0.979 (0.957–1.001)	0.060

*OR* odds ratio, *CI* confidence interval, *CKD* chronic kidney disease, *BMI* body mass index, *Hb* hemoglobin, *ALB* albumin, *TG* triglyceride, *PTH* parathyroid hormone, *SBP* systolic blood pressure, *DBP* diastolic blood pressure, *eGFR* estimated glomerular filtration rate, *LVEF* left ventricular ejection fraction

## Discussion

Pulmonary hypertension (PH) is a disease of the progressive condition characterized by elevated pulmonary arterial pressures and pulmonary vascular resistance leading to right ventricular failure [10]. The pulmonary vascular injury underlying PH occurs in an idiopathic form or in association with other disease states or exposures and is probably a final common response to environmental or disease-related inciting factors coupled with genetically determined susceptibilities [11]. Many studies have shown that PH occurred frequently in patients with chronic kidney disease (CKD), especially in end-stage renal disease (ESRD) patients. However, few epidemiologic data are available to investigate the prevalence of PH prior to CKD stage 4/5. In our study, we observed the prevalence of PH in different stages of CKD patients, including HD and PD patients, and investigated the relation between PH and CKD.

Some researches have reported that the prevalence of PH ranged from 9–39% in individuals with stage 5 CKD, 18.8–68.8% in hemodialysis patients, and 0–42% in patients on peritoneal dialysis therapy [3]. Wei Shi et al. reported that the prevalence of PH in CKD Stage 1-5D was 2.2, 6.7, 7.9, 15.2, 20.0 and 37.5%, respectively [12]. In our study, the overall prevalence of PH was 47.38% in the CKD population. The prevalence of PH in CKD stage 1–5 was 14.29, 33.33, 38.89, 40.91 and 64.47%. Prevalence estimates are variable due to several possible reasons. First, varying definitions of PH might account for varying prevalence estimates. Second, variable degrees of volume overload might also confound the prevalence. Third, some of CKD patients lack of echocardiography data were excluded.

We found that the prevalence of PH was increased with the deterioration of renal function, however, which had no direct relation to the severity of PH. Moderate-to-severe PH might occur in any stages of CKD, indicating that PH was a multi -factorial disorder in ESRD. However, we found that most of PH patients in CKD stage 1–2 were asymptomatic and had no records of any specific therapy for PH. Prostacyclin has been the mainstay of PH treatment for more than a decade. And it appeared to have cytoprotective and antiproliferative activities, which was also used for the treatment of CKD [10]. Therefore, the distribution of mild-moderate-severe PH in CKD stage 3–5 might be varied due to prostacyclin. The Doppler-estimated PASP was much higher in patients who received dialysis. It had no difference in the overall prevalence of PH between HD and PD patients. However, the distribution of moderate-to-severe PH varied differently in dialysis patients.

The baseline demographic, clinical, laboratory, and hemodynamic characteristics of patients with and without PH were observed. Associated determinants of PH in our study have been noted to be the following: higher BMI, lower hemoglobin, albumin and complement 3, increased TG, PTH and proteinuria, higher blood pressure, lower eGFR, lower LVEF and increased dialysis time. This implied that PH was determined by a diverse set of complex factors in CKD patients. Eventually, we found that BMI, hemoglobin, TG, proteinuria, PTH and eGFR were to be strongly associated with PH. BMI as a surrogate of body composition is highly correlated with overweight, unhealthy lifestyle and abnormal metabolism [13]. Obesity is risk factor for the development of cardiovascular disease, diabetes mellitus, renal diseases and some others [14]. Therefore, Higher BMI has been used to predict renal dysfunction, hypertension and metabolic abnormalities [15]. According to our study, it also demonstrated a positive relationship between BMI and PH. It has been proved that severe anemia, an established cardiovascular risk factor in CKD, may extend its impact to pulmonary circulation. Low hemoglobin levels may contribute to PH by aggravating hypoxia triggered by concomitant conditions [7, 16]. Right ventricular (RV) failure is the leading cause of death in PH and often progressed independent of the hemodynamic response to pulmonary vasodilators. Some researches have shown that circulating free fatty acids and long-chain acylcarnitines were significantly elevated in PH patients and increased in vivo myocardial triglyceride accumulation in PH [17]. Secondary hyperparathyroidism is a severe complication in CKD patients. Ulrich et al. reported that secondary hyperparathyroidism, which was not related to kidney disease but possibly to lower vitamin D status, was a risk factor for PH [18]. An elevated PTH level is associated with increased N-terminal pro-brain natriuretic peptide, a marker of left ventricular wall stress, and increased risk of heart failure [19]. Genctoy G et al. also confirmed that secondary hyperparathyroidism was associated with PH in older patients with CKD and proteinuria [20]. Consistent with our findings, PTH could contribute to PH in CKD patients. Amounts of studies have proved that immune dysfunction involved in the pathogenesis of renal disease, including pro-inflammatory monocytes, mast-cell proliferation, T-lymphocyte dysfunction and decreased T-regulatory cells, et al [21–24]. An increase in circulating inflammatory mediators also causes an increase in oxidative stress resulting in endothelial dysfunction [25]. Proteinuria in CKD is also a predictor of endothelial dysfunction and has a direct relationship with cardiovascular mortality [20]. Based on the action of the combination of factors, glomeruli repeat injury and repair constantly, which led to renal function deterioration eventually. Interestingly, endothelial dysfunction is a main trigger of pulmonary hypertension.

To be mentioned, some factors were not independent determinants of PH in the present study, however, they might be considered a “warning”. CKD is a progressive disease and involves alterations of etiologies, eg glomerulonephritis, diabetic nephropathy, hypertensive nephropathy, lupus nephritis, polycystic kidney disease, some of which associated with the prevalence of PH. Hypertension and diabetes mellitus trigger left ventricular diastolic dysfunction, an alteration bound to increase pulmonary venous and arterial pressure [3, 26]. In PH patients with systemic lupus erythematosus (SLE), macrophages, lymphocytes, antinuclear antibodies, and complement have been identified histologically in the pulmonary vasculature [27, 28]. Patients with diabetic nephropathy are more susceptible to mild PH and patients with lupus nephritis are more likely to suffer from severe PH compared to glomerulonephritis patients. The incidence of PH in patients with hypertensive nephropathy is not significantly increased due to well controlled blood pressure. In addition, the risk of PH in female patients is higher than male ones probably due to the serotonin, mutations in the bone morphogenetic protein receptor (BMPR) II gene, and estrogens [29].

In dialysis patients, the prevalence of PH was much higher, which had some specific risk factors for PH. In this study, patients with congenital heart failure and acute heart failure before had been excluded. However, subclinical heart failure was common in ESRD patients. The causes might include hypertension, salt and water overload, pleotropic effects of uraemic toxins and myocardial ischaemia. These factors were more prevalent in patients with PH. Arteriovenous fistulae (AVF) are considered the gold standard for HD access [30]. They result in increased venous return with a concomitant increase in cardiac output and also lead to decreased systemic vascular resistances [3, 25]. Well-performed studies show that AVF flow and AVF duration are related independently to the severity of pulmonary hypertension in HD patients [31]. In a study of patients receiving PD, LV mass index, alongside low serum albumin and fluid overload, was a predictor of PH in a multivariate model [32].

Our study still has several potential limitations. First, the diagnosis of PH was only based on echocardiography without right-sided heart catheterization (RHC), the gold standard diagnostic tool, to confirm the echocardiographic findings. Second, not all the CKD patients from July 2013 to September 2015 were enrolled in the study because some of them lacked the data of echocardiography. Third, the follow-up data to evaluate the effect of PH on morbidity and mortality were not available due to the retrospective study. Finally, this was a single center study. More data of multi-center needed to be included in to confirm the conclusions.

## Conclusions

Based on our study, it confirms that the prevalence of PH is increased with the deterioration of renal function, however, which has no direct relation to the severity of PH. PH occurs with more frequency in dialysis patients. However, it has no difference in the overall prevalence of PH between HD and PD patients. PH is determined by a diverse set of complex factors including BMI, hemoglobin, TG, proteinuria, PTH and eGFR in CKD patients. Therefore, higher BMI and TG, more sever anemia, proteinuria and secondary hyperparathyroidism, poor renal dysfunction predict the more prevalence of PH. Females, lupus nephritis and diabetic nephropathy could be considered as a “warning”. On the other hand, our findings further the epidemiology profile of PH across the spectrum of CKD.

## Abbreviations

AVF: Arteriovenous fistulae
BMI: Body mass index
BMPR: Bone morphogenetic protein receptor
CKD: Chronic kidney disease
eGFR: Estimated glomerular filtration rate
ESRD: End-stage renal disease
HD: Hemodialysis
LVEF: Left ventricular ejection fraction
PAP: Pulmonary arterial pressure
PASP: Pulmonary artery systolic pressure
PD: Peritoneal dialysis
PH: Pulmonary hypertension
PTH: Parathyroid hormone
PVR: Pulmonary vascular resistance
RHC: Right-sided heart catheterization
RV: Right ventricular
SD: Standard deviation
SLE: Systemic lupus erythematosus
TG: Triglyceride
